# Design of filtering cable with defected conductor layer

**DOI:** 10.1038/s41598-024-55736-9

**Published:** 2024-03-04

**Authors:** Yunan Han, Shuangqing Xiong, Chunyue Cheng, Zhaohan Liu

**Affiliations:** 1 Department of the College of Information Science and Technology, Beijing University of Chemical Technology, Beijing, 100029 China; 2 Beijing Institute of Radio Metrology and Measurement, Beijing, 100854 China

**Keywords:** Electrical and electronic engineering, Electronics, photonics and device physics

## Abstract

Electrical cables, often referred to as ‘blood vessels’ and ‘nerves’ of the industry, play a vital role in the connection of electrical devices. However, traditional cables that lack distributed filtering functions are usually the primary coupling path for electromagnetic compatibility (EMC) problems. An innovative design for a filtering cable, which incorporates insulated electrical wires coated with a specific defected conductor layer (DCL), enables it to achieve distributed filtering advantages along its axis. Microwave network analysis is employed to build the two-port network model of filtering cable, which efficiently analyzes the cascading characteristics of periodic or aperiodic filtering cables. To validate, the flexible printed circuit board (FPCB) with sawtooth dumbbell-shaped DCL and mounted by capacitors is wrapped around the stripped section of the coaxial cable to manufacture a multi-stopband filtering cable. Simulated and measured results demonstrate that the proposed filtering cable can be effectively suppressed in the stopband, which can be adjusted by changing the values of capacitors.

## Introduction

Electrical cables, composed of one or more wires that may be insulated and shielded^1^, play a crucial role in transmitting electrical power^2^ or signals^3,4^ between different equipment or modules. They are widely utilized in various industries, earning them the epithets of ‘blood vessels’ and ‘nerves’ of the industrial realm, indicating their indispensable importance in driving global economic advancement. However, electrical cables, as current-carrying conductors, are usually the coupling path of electromagnetic compatibility (EMC) issues^5^. They may not only conduct electromagnetic interference (EMI), resulting in symptoms of conducted emissions (CE) and conducted susceptibility (CS)^6^, but also radiate electromagnetic energy, giving rise to problems with radiated emissions (RE) or radiated susceptibility (RS)^7^. These effects are generally undesirable and can easily lead to electrical equipment malfunction^8^. With the rapid expansion of transformative technologies such as smart grid, next-generation mobile communication, the internet of things^9^, unmanned platforms, and artificial intelligence, traditional electrical cables face significant challenges in terms of EMC^10–15^.

In order to improve EMC, shielded cables and lumped filters are commonly employed for interconnections within and between equipment. Existing particular cables and filters technologies include shielding^16^, coaxial geometry for achieving electromagnetic shielding with stable impedance and phase^17^, twisted-pair geometry for reducing common-mode interference^18^, flexible microstrip filter transmission line with defected ground structure (DGS)^19^, suspension filter circuit^20^, filtering connector^21^, magnetic material filtering^22^, lumped electronic components filters^23^, waveguide filters^24^, ceramic filters^25,26^, DGS filters^27^, microstrip filters^28,29^, and various types of filters^30,31^, etc. However, it is worth noting that, currently there is a lack of distributed filtering functionality along the length of the cable.

In this work, we present a filtering cable with distributed filtering functions, introduce its design method, and manufacture a multi-stopband filtering cable using sawtooth dumbbell-shaped defected structures (SDDSs) and surface-mounted (SMD) capacitors on a defected conductor layer (DCL) for verification. From a field perspective, the DCL-based filtering cable can be regarded as a waveguide with complex boundaries. For electromagnetic waves in different frequency bands, a guided traveling wave is formed in the passband and a backward wave is formed in the stopband. The DCL-based filtering cable can be simulated using commercial full-wave simulation software such as HFSS^32^. However, for a long filtering cable, the simulation consumes a large amount of computing resources, which brings great inconvenience to the design. A more feasible scheme is to obtain the *ABCD* matrix of each shorter resonant unit of the filtering cable through simulation and calculate the *ABCD* matrix of the entire filtering cable by cascading the two-port network model of the filtering cable units to achieve the transmission parameters of the entire filtering cable. From a circuit point of view, the defected conductor layer of the DCL-based filtering cable can be converted into a circuit model consisting of capacitors and inductors, incorporated into the cable circuit to suppress EMI signals. To validate the calculation and simulation method, we conducted a study on a multi-stopband filtering cable that incorporates sawtooth dumbbell-shaped structures on the DCL. Through this study, we can summarize the underlying patterns within its transmission characteristics.

## Methods

### Filtering cable structure design

By using specific DCL to replace part of the shield of the coaxial cable, a filtering cable with a multi-stopband filtering function can be designed. The DCL manufactured by a FPCB has the advantages of being light, low water absorption, and inexpensive. The SDDS on DCL combined with the capacitor at the gap of the SDDS can make the filtering cable with adjustable band rejection characteristics.

### Cascade analysis of two-port network model of filtering cable

On the basis of microwave network analysis theory, a two-port network model of the filtering cable is constructed. When the coupling between filtering cable units is very weak, the S-parameters of a single filtering cable resonator are simulated by full-wave simulation, and the S-parameters of periodic or aperiodic filtering cable cascade structure can be obtained by cascade analysis of the two-port network model, which saves the time of simulating the ultralong filtering cable.

### Full-wave simulation and measurement

For the design and simulation of the proposed filtering cable and each of the resonate units, the high frequency structure simulator (HFSS) is used. The S-parameters and radiation of the proposed filtering cables are measured using the Keysight network analyzer (PNA N55524B calibrated by N4691) in the anechoic chamber.

### Basic structure design

**Figure 1:**
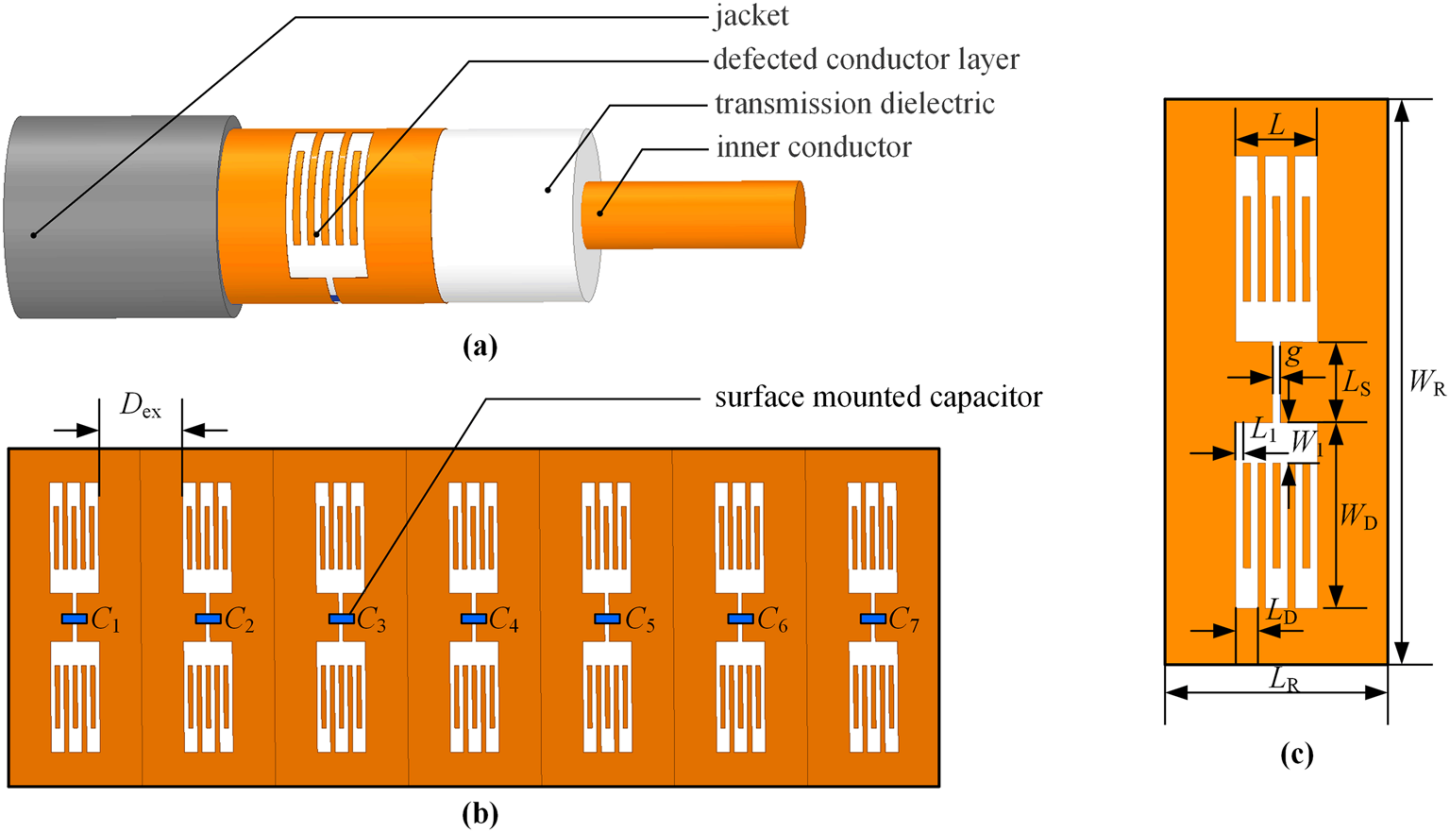
The proposed multi-stopband filtering cable with 7 sawtooth dumbbell-shaped resonators on DCL. (**a**) The characteristic filtering cable configuration. (**b**) The unfolding DCL of the designed rejection band filtering cable. (**c**) The unfolding DCL features an individual sawtooth dumbbell-shaped defected structure.

Illustrated in Fig. 1a, the filtering cable featuring a suspended circuit showcases a geometric structure comprising an inner conductor, a transmission dielectric, a defected conductor layer (DCL) with integrated components, and a jacket, arranged from the innermost to the outermost layer. The proposed filtering cable structure bears a resemblance to that of coaxial cables, albeit with a distinguished distinction in the outer shielding layer. Instead of encompassing a complete structure, it showcases meticulously etched patterns and surface-mounted electronic components.

As illustrated in Fig. 1b, the DCL has etched periodic or aperiodic defected structures, accompanied by strategically positioned surface-mounted components on the conductor. In this particular case, the unwrapped DCL gracefully embraces seven periodic SDDSs, accentuated by the placement of SMD capacitors within the core of the exquisite sawtooth dumbbell-shaped formations, bridging the divide amidst the defected structures. The unfolding DCL features an individual sawtooth dumbbell-shaped defected structure, as shown in Fig. 1c.

### Two-port network modeling of the filtering cable

**Figure 2:**
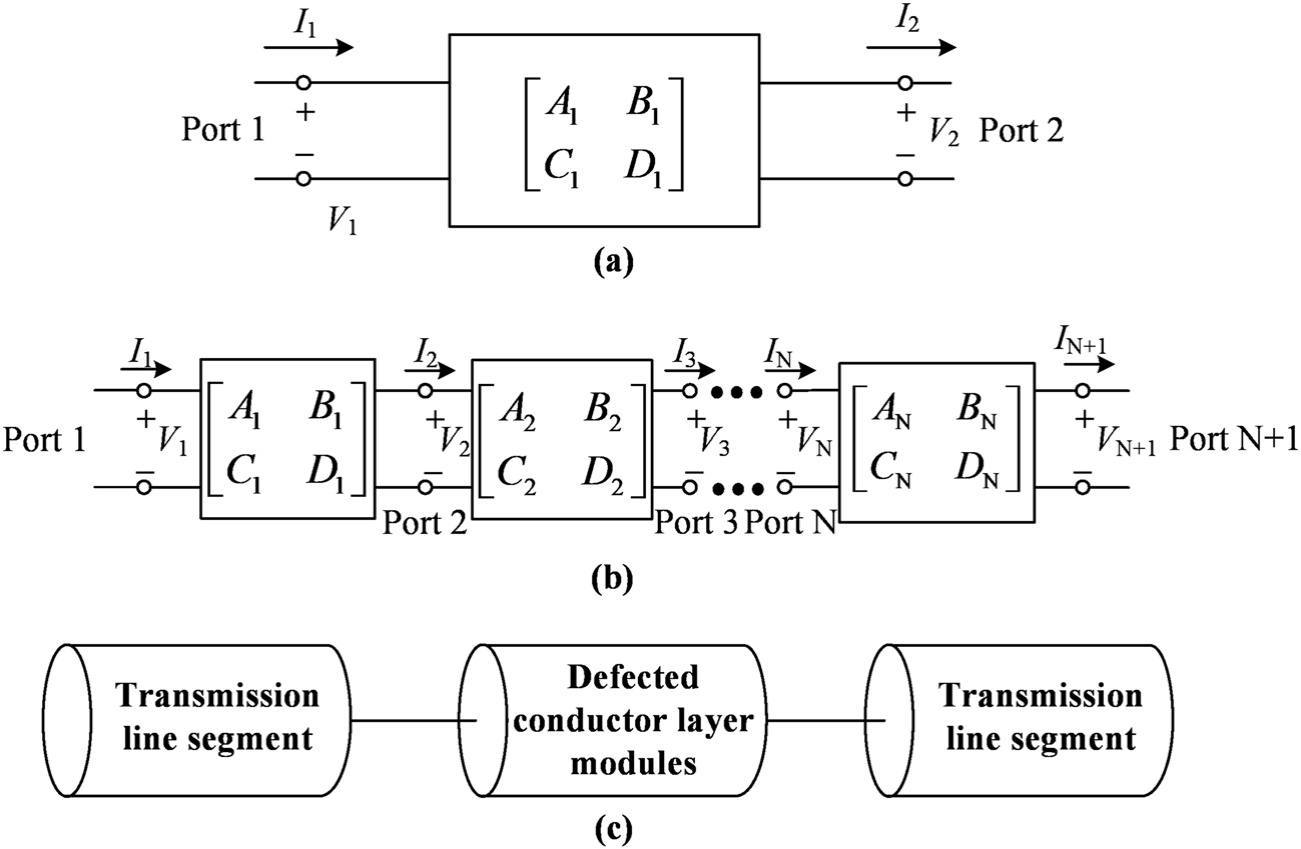
The two-port network of the DCL-based filtering cable. (**a**) The two-port network of the whole DCL-based filtering cable. (**b**) Two-port series networks of the DCL-based filtering cable composed of N resonant sections. (**c**) The axial structural decomposition of the resonator of the DCL-based filtering cable.

We use microwave network analysis to model the two-port network of the filtering cable. The transmission performance of the DCL-based filtering cable can be modeled as the *ABCD* matrix of a two-port network in terms of the total voltages and currents, as shown in Fig. 2a and the following^33^:

1 $$\left[ {\begin{array}{*{20}c} {V_{1} } \\ {I_{1} } \\ \end{array} } \right] = \left[ {\begin{array}{*{20}c} {A_{{1}} } & {B_{{1}} } \\ {C_{1} } & {D_{1} } \\ \end{array} } \right]\left[ {\begin{array}{*{20}c} {V_{2} } \\ {I_{2} } \\ \end{array} } \right]$$

Similar to a coaxial cable, the output voltage and current characteristics of the filtering cable can be obtained from the input voltage and current in the required frequency band.

In addition, the filtering cable with *N* resonant sections can be equivalent to the cascade connection of *N* two-port networks, equivalent to its various parts as shown in Fig. 2b, in case the coupling between the resonant sections of the filtering cable can be ignored. Under this premise, the transmission parameters of the filtering cable can be calculated by multiplying the cascaded *ABCD* matrices of *N* two-port networks, which can be easily obtained separately by the full-wave simulation of each resonant section. In the cascade connection of *N* two-port networks, we have

2 $$\left[ {\begin{array}{*{20}c} {V_{1} } \\ {I_{1} } \\ \end{array} } \right] = \prod\nolimits_{{{\text{i}} = 1}}^{{\text{N}}} {\left[ {\begin{array}{*{20}c} {A_{{\text{i}}} } & {B_{{\text{i}}} } \\ {C_{{\text{i}}} } & {D_{{\text{i}}} } \\ \end{array} } \right]} \left[ {\begin{array}{*{20}c} {V_{{{\text{N}} + 1}} } \\ {I_{{{\text{N}} + 1}} } \\ \end{array} } \right]$$

which shows that the *ABCD* matrix of the cascade connection of the two networks is equal to the product of the *ABCD* matrices representing the individual two ports.

The *ABCD* matrix for each resonant section can be studied in more detail. For the filtering cable resonator with the specific etched-out pattern on DCL, it can be modeled into two-port networks, including two classical transmission sections at both ends and a defected conductor section in series along the axial direction as shown in Fig. 2c. In general, the etched motif on the DCL displays periodicity, enabling the formation of a distributed circuit arranged in series and its incorporation into the circuit framework of a classical transmission line.

With simulation by HFSS, the equivalent circuit model of a specific defected resonant unit is mathematically treated as a transfer *ABCD* matrix:

3 $$ABCD_{{{\text{DC}}}} = \left[ {\begin{array}{*{20}c} {A\left( {R,L,C} \right)} & {B\left( {R,L,C} \right)} \\ {C\left( {R,L,C} \right)} & {D\left( {R,L,C} \right)} \\ \end{array} } \right]$$

In the equivalent circuit model, *R* is the resistance, *C* is the capacitance, and *L* is the inductance. That is to say, each element in the *ABCD* matrix is related to the equivalent circuit of the filtering cable resonator and can be obtained by full-wave simulation.

The *ABCD* matrix for the resonant section of the filtering cable can be calculated by simulated S-parameters as^33^

4 $$\left[ {\begin{array}{*{20}c} A & B \\ C & D \\ \end{array} } \right] = \left[ {\begin{array}{*{20}c} {\frac{{\left( {1 + S_{11} } \right)\left( {1 - S_{22} } \right) + S_{12} S_{21} }}{{2S_{21} }}} & {Z_{0} \frac{{\left( {1 + S_{11} } \right)\left( {1 + S_{22} } \right) - S_{12} S_{21} }}{{2S_{21} }}} \\ {\frac{1}{{Z_{0} }}\frac{{\left( {1 - S_{11} } \right)\left( {1 - S_{22} } \right) - S_{12} S_{21} }}{{2S_{21} }}} & {\frac{{\left( {1 - S_{11} } \right)\left( {1 + S_{22} } \right) - S_{12} S_{21} }}{{2S_{21} }}} \\ \end{array} } \right]$$

For the coaxial transmission section, the *ABCD* matrix is^31^

5 $$ABCD_{{\text{T}}} = \left[ {\begin{array}{*{20}c} {{\text{ch}} \left( {\gamma d} \right)} & {Z_{0} {\text{sh}} \left( {\gamma d} \right)} \\ {\frac{{{\text{sh}} \left( {\gamma d} \right)}}{{Z_{0} }}} & {{\text{ch}} \left( {\gamma d} \right)} \\ \end{array} } \right]$$

where *γ* is the transmission constant, *d* is the actual physical length of the transmission line segment, and *Z*_0_ is the characteristic impedance of the coaxial transmission line.

After cascading classical transmission lines at both ends of the filtering cable resonant unit, the *ABCD* matrix of a defected conductor resonant section can be obtained:

6 $$ABCD_{{\text{R}}} = ABCD_{{\text{T}}} \cdot ABCD_{{{\text{DC}}}} \cdot ABCD_{{\text{T}}}$$

In the case of weak coupling of each resonator, the *ABCD* matrix of N multiple resonators can be obtained by cascading:

7 $$ABCD_{{\text{N}}} = \prod\limits_{{{\text{i}} = 1}}^{{\text{N}}} {ABCD_{{{\text{Ri}}}} }$$

Substitute Eq. (7) into Eq. (4) to reverse the corresponding S matrix, and the transmission characteristics of the DCL-based filtering cable can be obtained.

Using the above method, the entire S-parameters of the filtering cable can be obtained by simulating the S-parameters of the filtering cable unit and then cascading them through two-port networks. This approach can significantly reduce the design time required for the filtering cable.

### Manufacture

**Figure 3:**
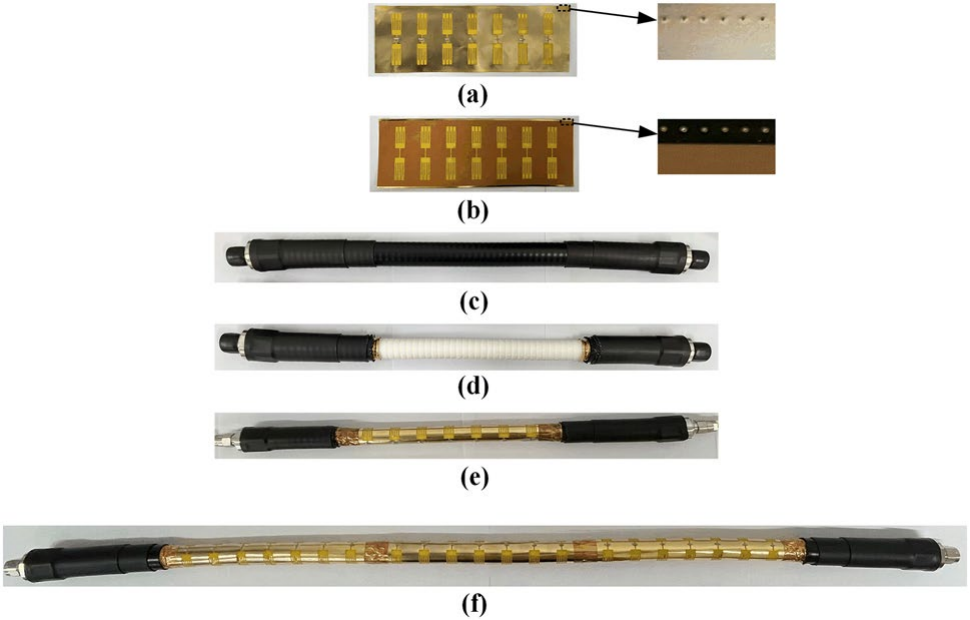
Photographs of the proposed multi-bandstop filtering cable fabricated with SDDSs on the defected conductor layer. (**a**) The unfolding DCL with 7 cascaded resonant units (top side). (**b**) The unfolding DCL with 7 cascaded resonant units (bottom side). (**c**) The Andrew AVA5-50 coaxial cable with a length of 0.5 m. (**d**) The 0.5 m coaxial cable with part of the jacket layer and shielding layer removed. (**e**) The proposed 0.5 m filtering cable with 7 cascaded resonators. (**f**) The proposed 1 m filtering cable with 21 cascaded resonators.

The filtering cable featuring a defected conductor layer can be manufactured using well-established techniques. DCLs are manufactured using a FPCB, which has excellent flexibility, allowing it to bend into a cylindrical shape, while having the advantages of low water absorption, being lightweight, and being low cost. As depicted in Fig. 3a, the DCL can be fabricated using an 18-µm-thick gold-plated copper sheet on a FPCB. The copper sheet consists of 7 periodic cascaded sawtooth dumbbell-shaped etched-out patterns. Each bridged part of the SDDS is welded with a SMD capacitor. The exact capacitance values of the SMD capacitors can be measured using a digital bridge tester, and the capacitance of the capacitors increases from left to right in order. Fig. 3b shows the bottom side of the FPCB, which has a polyimide (PI) film substrate with a thickness of 0.13 mm, a relative permittivity of 3.1, and a dielectric loss tangent of 0.0028. The dimensions of the FPCB are 230 mm × 77.8 mm × 0.148 mm. There are 1 mm wide conductors on the upper and bottom surfaces around the FPCB, and they are connected through periodic vias, ensuring a good connection at the junction when the FPCB is rolled onto the transmission dielectric.

As shown in Fig. 3c, the Andrew AVA5-50 type coaxial cable is chosen as the foundation for refitting the filtering cable, with lengths of 0.5 m and 1 m, respectively. The coaxial cable features a copper wire with a diameter of 9.45 mm, corresponding to the inner conductor of the proposed filtering cable. Foam polytetrafluoroethylene (PTFE) is used as the material for the transmission dielectric, with a relative permittivity (*ε*_r_) of 1.27, a dielectric loss tangent of 0.001 and an outer diameter of 24.13 mm. Take the 0.5 m long filtering cable as an example, the filtering cable can be obtained by stripping the jacket and shielding layer of the middle part of the coaxial cable, as shown in Fig. 3d, wrapping the FPCB of the defected conductor layer, and welding with the shielding layer at both ends. The filtering cable, which is 0.5 m long and constructed in this manner, is depicted in Fig. 3e. Similarly, the 690-mm long outer conductor of the shielding layer is removed from the 1 m long coaxial cable, and three identical sets of flexible circuit boards, soldered with surface-mounted capacitors, are wrapped around the outer surface of the dielectric layer, as illustrated in Fig. 3f. The capacitance of each set of seven capacitors increases from left to right, as shown in Fig. 1b. The capacitance values of the three sets of capacitors are the same in the corresponding positions. The shortest distance between the defected parts of two resonant units is *D*_ex_ = 19 mm. The capacitance values of seven SMD capacitors are *C*_1_ = 69.4 pF,* C*_2_ = 82.5 pF, *C*_3_ = 100.8 pF,* C*_4_ = 119.3 pF,* C*_5_ = 153.2 pF,* C*_6_ = 198.4 pF, and *C*_7_ = 399.4 pF, respectively. Fig. 1c illustrates the unfolding DCL with an individual sawtooth dumbbell-shaped resonator. The structural parameters of the DCL are *L*_R_ = 30 mm, *W*_R_ = 72.4 mm,* L* = 11 mm, *L*_D_ = 3 mm, *W*_D_= 24.97 mm,* L*_S_ = 10.86 mm,* L*_1_ = 1 mm, *W*_1_ = 5.43 mm, and *g* = 1mm, respectively.

## Results and discussion

### Simulation and measurement

**Figure 4:**
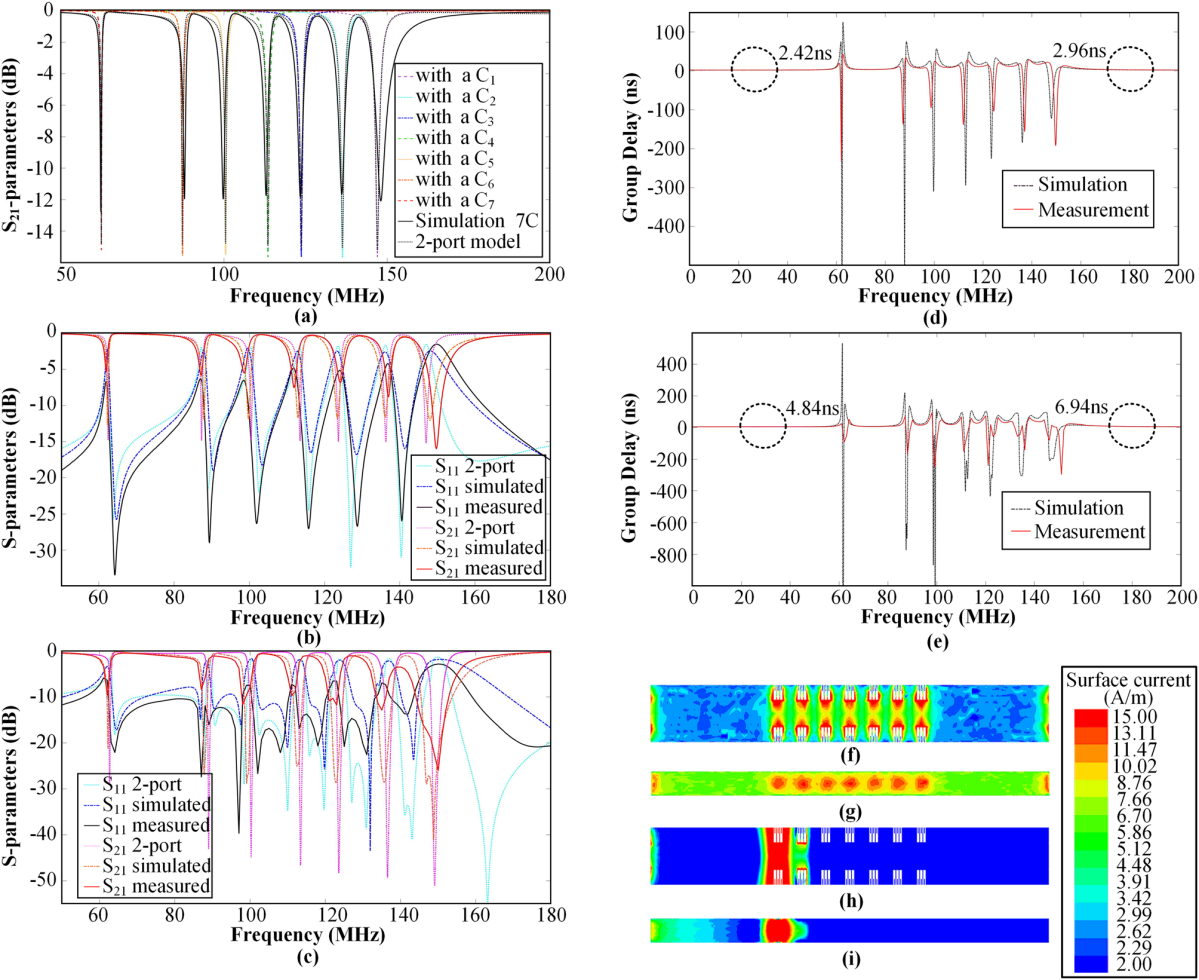
Simulation and measurement results of the filtering cable based on sawtooth dumbbell-shaped DCL. (**a**) Comparison of S_21_-parameters between an individual resonator with seven different capacitances and a cascade of seven resonators with different capacitors. (**b**) The S-parameters for the filtering cable with 7 cascaded resonators with a length of 0.5 m. (**c**) The S-parameters for the filtering cable with 21 cascaded resonators with a length of 1 m. (**d**) The group delay for the 0.5-m-long filtering cable with 7 cascaded resonators. (**e**) The group delay for the 1-m-long filtering cable with 21 cascaded resonators. (**f**) Surface current distribution on the DCL at 10 MHz within the passband. (**g**) Surface current distribution on the inner conductor at 10 MHz within the passband. (**h**) Surface current distribution on the DCL at 148 MHz within the stopband. (**i**) Surface current distribution on the inner conductor at 148 MHz within the stopband.

As depicted in Fig. 4a, the variation of S-parameters with capacitances can be obtained through simulation when seven different capacitances of the SMD capacitor of the filtering cable resonator are separately taken. As the capacitance of the chip capacitor increases, the resonant frequency point corresponding to the S_21_ curve moves towards a lower frequency. Specifically, as the chip capacitance increases from 69.4 to 399.4 pF, the resonant frequencies of the filtering cable resonant unit are 147 MHz, 136.29 MHz, 123.69 MHz, 113.49 MHz, 100.53 MHz, 87.33 MHz, and 62.46 MHz, respectively. According to the variation rule of these seven resonant frequencies with these capacitance values, we derive the following equation:

8 $$f = \frac{1236}{{\sqrt C }}$$

where* C* is the capacitance value of the surface-mounted capacitor in pF; *f* is the resonance frequency of the filtering cable resonator in MHz. Based on the required filtering frequency in the range of 50–150 MHz, the corresponding capacitance value can be calculated by using this equation. This allows the bandstop characteristics of the filtering cable with the surface-mounted capacitor taking different capacitance values to be verified.

Using the two-port network model of filtering cable, the *ABCD* matrix corresponding to each of the seven resonant units can be extracted according to the S-parameter matrix obtained from the simulation. These matrices undergo a two-port network cascade operation to obtain the *ABCD* matrix for the cascade structure of the seven resonant units, enabling the calculation of the theoretically predicted S-parameter transmission characteristics for the cascaded structure of the filtering cable. This demonstrates the applicability of the two-port network model to the cascade characteristics of filtering cables based on the DCL of a sawtooth dumbbell structure. Figure 4a illustrates the S-parameter curves for a cascade of seven different resonant units compared with an individual resonator with seven different capacitors. The resonant frequency of the S_21_ curve, obtained by analyzing the cascade of resonant units using a two-port network model with seven different capacitors, closely corresponds to the resonant frequency of a single resonator using seven different capacitors. However, the stop-band suppression is not as high as that. Additionally, the full-wave simulation closely aligns with these frequencies. Any slight deviation in the resonant frequency can be primarily attributed to the coupling effect between the different units that occurs after the cascade of resonators.

Figure 4b illustrates a comparison of the S-parameters obtained through various methods, including the two-port network model, full-wave simulation using HFSS, and experimental measurement. The S-parameters of the proposed 0.5-meter-long filtering cable are measured using the Keysight network analyzer (PNA N55524B calibrated by N4691). In Fig. 4b, the resonant frequencies of the three S_21_ curves are closely aligned, indicating the applicability of the two-port network model in microwave network analysis for cascaded filtering cables. The slight deviation between the simulated and measured resonance frequencies can be attributed to manufacturing errors in structural processing and the influence of connectors. The two-port network model achieves significantly higher stopband suppression at each resonant frequency, resulting in an extremely narrow stopband bandwidth. However, both the simulated and measured values exhibit a lower insertion loss compared to the theoretical values, except for the maximum resonant frequency point. Although efforts have been made to minimize the coupling effect between the cascaded filtering cable resonant units, it cannot be completely eliminated. The microwave network analysis using the two-port matrix operation model does not account for the coupling effect between different resonant units. The measured |S_21_| at the highest resonant frequency point outperforms the simulated value, while the measured |S_21_| at the remaining frequency points is lower than the simulated value.

The insertion loss S_21_ and return loss S_11_ of the 1-meter-long filtering cable, which consists of 21 resonant units cascading as shown in Fig. 3f, are compared in Fig. 4c. The results are obtained using various methods, including the two-port network model, full-wave simulation using HFSS, and experimental measurement. The resonant frequencies obtained from these three methods are found to be in close agreement. When compared to the case of a group of seven cascaded resonant units, the insertion loss (|S_21_|) at each resonant frequency point for the cascading of the three-set defective conductor layers is observed to increase by nearly 5 dB. This indicates that the cascaded periodic resonant units can effectively improve the rejection in the stopband. Therefore, cascading multiple sets of defected resonance units can achieve a significant enhancement in the filtering function for signals at specified frequency points. Furthermore, compared to the case of seven cascaded resonant units, the actual measured resonant frequency points for the filtering cable with a cascade of 21 resonant units show an increase in the stopband bandwidth.

This increase is attributed to factors such as deviations in the capacitance value of each group and the coupling effect between the resonant units. In conclusion, the results demonstrate that the proposed cascading technique using multiple sets of defected resonance units is effective in achieving improved rejection in the stopband and expanding the stopband bandwidth.

The frequency-dependent group delay of the interconnected configuration consisting of seven resonant units in the 0.5-meter-long filtering cable has been both simulated and measured, as visually illustrated in Fig. 4d. It should be noted that the simulated and measured group delay curves demonstrate remarkable concurrency, showcasing their high level of agreement. Within the passband range, the group delay of different frequency signals remains relatively constant, measuring less than 3 ns. The average group delay to the left of the first resonant frequency point is 2.42 ns, whereas to the right of the seventh resonant frequency point, it is 2.96 ns. These findings indicate that the filtering cable can effectively transmit passband frequency signals within the 0–200 MHz range. Similarly, the simulated and measured group delay curves of the 3-group cascade structure 1 m long filtering cable are presented in Fig. 4e. The simulated and measured curves exhibit strong agreement within the passband with the group delays of different frequency signals in the passband that remain relatively consistent and below 10 ns. The average group delay to the left of the first resonant frequency point is 4.84 ns, while to the right of the seventh resonant frequency point, it is 6.94 ns. These results further validate the filtering cable’s ability to ensure the unobstructed transmission of passband signals. The surface current distribution of the inner conductor and sawtooth dumbbell-shaped DCL of the 0.5m-long filtering cable, as depicted in Fig. 3e, is simulated using HFSS. Fig. 4f shows the surface current distribution of the DCL of the filtering cable, while Fig. 4g illustrates the surface current distribution of the inner conductor at a frequency of 10 MHz within the passband. In the passband, the current exhibits a smooth distribution on the surface of both the inner and outer conductors, indicating the normal transmission of signals through the filtering cable. At a frequency near the seventh resonant frequency, such as 148 MHz, the surface current distribution of the DCL of the filtering cable is obtained as shown in Fig. 4h, while Fig. 4i presents the surface current distribution of the inner conductor. Near the seventh resonant frequency, the current becomes concentrated at the resonant unit of the filtering cable, which determines the functional location of this resonant frequency. This concentration of current signifies that signals within the stopband range are slightly passing through the filtering cable, demonstrating its effective distributed filtering function.

**Figure 5:**
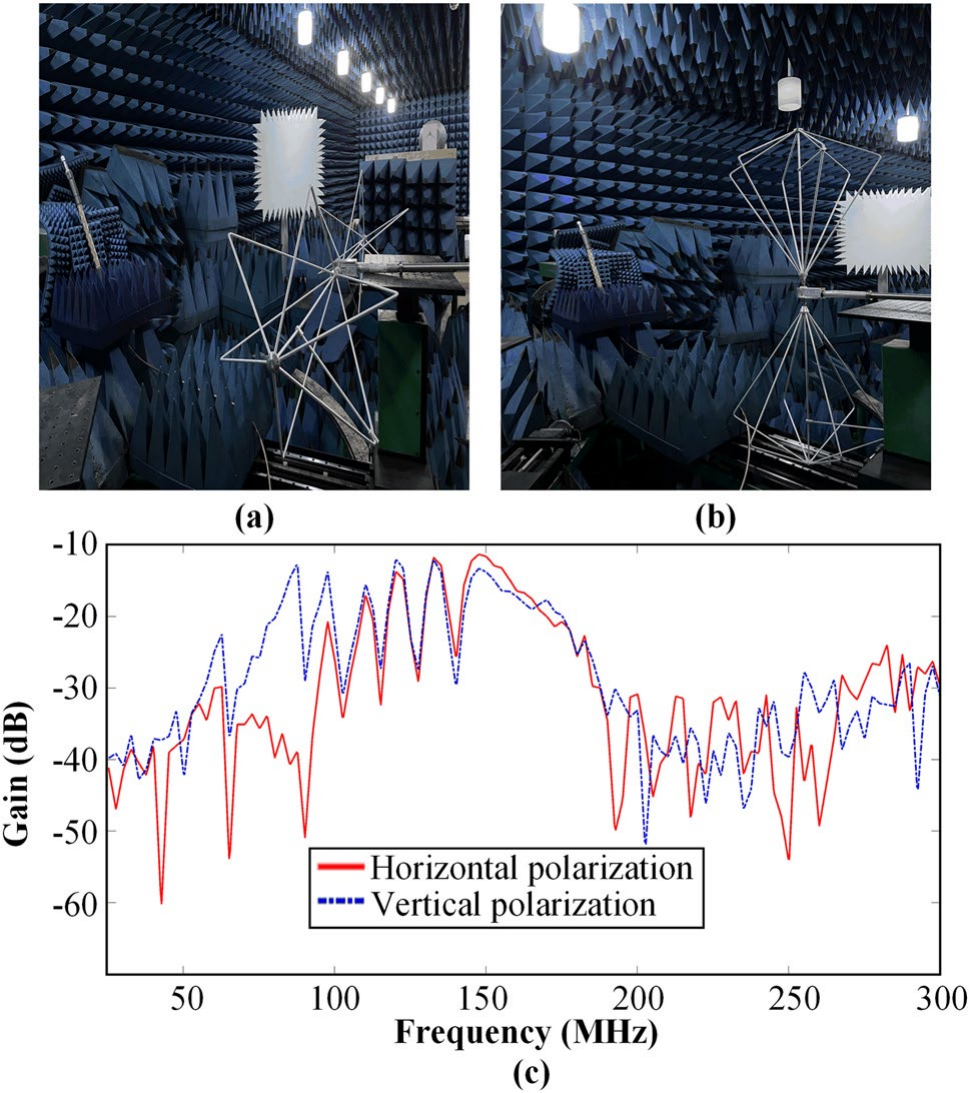
Measurement of the radiation performance of 0.5-m-long filtering cable in the anechoic chamber. (**a**) The biconical antenna is horizontally polarized. (**b**) The biconical antenna is vertically polarized. (**c**) Variation of the filtering cable gain with frequency.

Using HFSS to simulate the 0.5 m long filtering cable, the radiated power at the highest resistance band frequency point of 148 MHz is only 3.29% of the input power, and the maximum gain of the radiation pattern is less than − 1.2 dB, indicating the filtering cable has extremely low radiation efficiency. According to the antenna radiation theory, the radiation efficiency of other lower frequencies of this filtering cable is even lower. In order to further analyze the radiation performance of the filtering cable, we have a radiation performance test in the anechoic chamber, as shown in Fig. 5. Specifically, for the test arrangement, port 1 of the vector network analyzer is connected to the filtering cable which the other end is connected with a 50-ohm load, and port 2 is connected to a VHBB 9124 model biconical antenna by SCHWARZBECK. We record the S_21_ of the vector network analyzer and further analyze it to obtain the gain versus frequency of the filtering cable. The placement of the filtering cable in Fig. 5a,b is kept constant, that is, the side of the soldered capacitors is facing the antenna, and the biconical antenna is placed horizontally polarized in the manner of Fig. 5a and vertically polarized in the manner of Fig. 5b, respectively, to obtain the curves of the gain versus frequency, as shown in Fig. 5c. The gain of the filtering cable in the range of 25–300 MHz is less than − 10 dB, and the maximum gain of the filtering cable is measured to be − 11.37 dB corresponding to 148 MHz. It can be seen from the simulation and measurement that there is basically no radiation effect in the proposed filtering cable operating below 200 MHz. The radiated power of the Andrew AVA5-50 type coaxial cable utilized in the fabrication of the filtering cable is less than 0.7% of the incident power below 300 MHz as per simulation results. Obviously, the maximum radiation gain of the coaxial cable is less than − 21.5 dB. Although the radiated power of the filtering cable is slightly higher compared to the coaxial cable used, it remains within an acceptable range for most application. In contrast to coaxial cables, filtering cables not only transmit signals but also provide a distributed filtering function.

**Figure 6:**
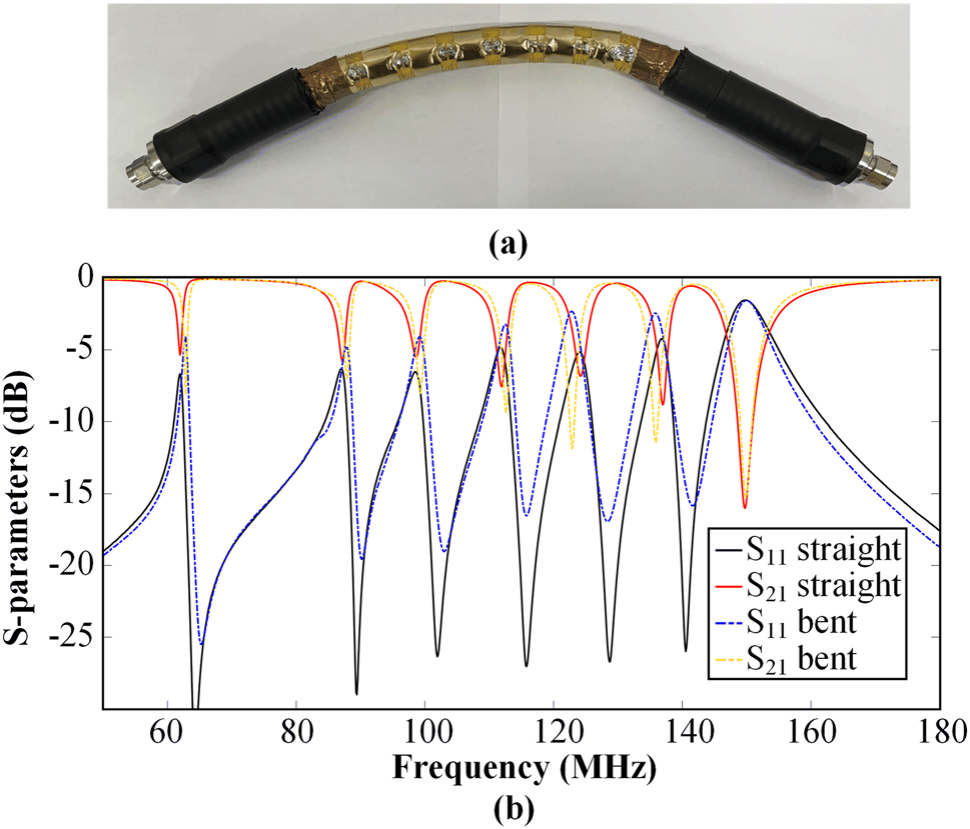
The S-parameter measurements of the 0.5 m long filtering cable when it is bent. (**a**) The filtering cable bent into a partial arc of a circle with a diameter of 1 m. (**b**) The comparison of measured S-parameters between the bent and straight filtering cables.

To investigate the transmission performance of the DCL-based filtering cable under bending conditions, we conducted an experiment where a 0.5 m long filtering cable was bent into a partial arc of a circle with a diameter of 1 m, as depicted in Fig. 6a. Using a vector network analyzer, we measured the S-parameters of the bent filtering cable. The comparison of measured S-parameters between the straight and bent filtering cables is presented in Fig. 6b. Notably, both S_21_ and S_11_ curves exhibit a high degree of overlap, indicating that the proposed filtering cables maintain a consistent filtering performance even when subjected to bending.

The developed filtering cable cascaded two-port network model is found to be of practical value through physical measurements. The model can be used to efficiently achieve the prediction of transmission characteristics of exceedingly long filtering cables for practical engineering applications, providing an important reference for further filtering cable cascade model design analysis.

### Performance analysis and comparison

The comparative characterization of the proposed filtering cables with various existing cables and filtering techniques is presented in Table 1. Shielding cables^16^ can shield EMI, coaxial cables^17^ can achieve high bandwidth communication over relatively long lines, and twisted wire pair^18^ can eliminate common-mode interference. However, none of them have filtering capabilities, while filtering cables are capable of filtering EMI.

**Table 1:** A comparative analysis of DCL-based filtering cables against other microwave devices.

References	Name	Typical structure	Characterization
This paper ^13^	DCL-based filtering cable		DCL-based filtering cable can be manufactured by wrapping a specific FPCB around an inner conductor. Shielding, armor, and grounding can also be applied outside as in a traditional cable. It is an efficient and complex electromagnetic environment solution that integrates filtering, shielding, and grounding. The most important feature is the distributed filtering function along the length of the cable
^14^	Shielding cable		Cables with a shielding layer can achieve electromagnetic shielding performance, but they do not have a filtering function
^15^	Coaxial cable		Invented by Oliver Heaviside, coaxial cables provide stable impedance and phase in a broad frequency band and have good signal integrity, but they do not have a filtering function
^16^	Twisted-wire pair		A pair of mutually insulated wires is twisted together to transfer differential mode signals and eliminate common mode interference
^17^	Flexible microstrip-filtered transmission line		The flexible microstrip-filtered transmission line is made of FPCB, which is very thin and light, and has filtering capabilities. It can replace traditional coaxial lines and be conveniently used for mobile or flexible communication terminals
^18^	Suspension filter circuit		The filtering function is achieved by adding capacitors and wires to the silicon rubber pad, which can be installed in the connectors. However, the filtering performance is weak and the reliability is not very high
^19^	Filtering connector		Filtering connectors can be achieved by adding capacitors and inductors inside the connector, which is a type of lumped filter rather than a distributed filter
^20^	Ferrite magnetic ring		Passing the cable through the magnetic ring can suppress high frequency interference on the cable. However, the disadvantage is that it is heavier and cannot be bent easily
^21^	Passive Power filter		The passive EMI power filter is composed of capacitors, inductors, and resistors. It has the advantage of being high-reliability and effective in solving EMI problems in the low frequency band. However, its disadvantage is that it has a large volume and heavy weight
^22^	Waveguide filter		Metal cavity filters have a very low insertion loss and high quality factor. However, their disadvantages are their large size, bulky nature, and high cost
^23,24^	Low-temperature co-fired ceramic (LTCC) filter		LTCC multilayer filters, commonly used in mobile communication terminals, have the advantage of being extremely small in size. However, their disadvantage is that they have a low quality factor and high insertion loss in the passband
^25^	Defected ground structure filter		Defected ground structure filters consist of a transmission line on one side of the substrate and a ground with a specific etching pattern on the other side of the substrate. This configuration can be equivalent to a filter circuit

Flexible microstrip-filtered transmission lines^19^ using FPCBs are thin, lightweight, and flexible compared to rigid defective ground filters^27^. Therefore, FPCBs are applied to the outer conductor of filtering cables with easy processing and desirable performances. Suspended circuit filters^20,21^ have poor filtering performances and low reliabilities. The ferrite magnetic ring^22^ can suppress high-frequency interferences, but it is heavy and not easy to be bent. The waveguide filter^24^ has low insertion loss and high-quality factor, but is large, bulky, and costly. The low-temperature co-fired ceramic (LTCC) filter^25,26^ is small in size but has a low-quality factor and poor passband insertion loss. EMI power filters employing lumped filtering circuits are primarily utilized to suppress conducted EMI within the 1-30 MHz range in power systems. These filters are broadly categorized into passive and active power filters. Passive power filters^23^ are characterized by their bulky size, substantial weight, and the utilization of costly inductors. Conversely, active power filters exhibit a more complex structure and their circuit stability is susceptible to environmental factors. Devices using these filtering techniques are either bulky or lumped filters, whereas the proposed filtering cable is lightweight and has distributed filtering effects. DCLs formed by FPCBs and capacitors provide a suspended filtering circuit, offering a flexible and cost-effective solution. This configuration contributes to the achievement of a stable filtering performance for the filtering cable.

## Conclusions

This paper presents a design and implementation method for filtering cable, focusing on a multi-stopband filtering cable example based on the defected conductor layer with sawtooth dumbbell-shaped defected structures and surface mounting capacitors. The transmission characteristic curves obtained from the two-port network model, full-wave simulation using HFSS, and experimental measurements exhibit a high level of consistency. This validates the applicability of the two-port network model from the microwave network analysis to the design of cascading characteristics for the filtering cable’s resonant units. The proposed filtering cable cascade model significantly reduces the simulation time required for analyzing the cascading characteristics of the filtering cable’s resonant units. Similar to how a musician composes a beautiful melody using the seven notes of a major scale, engineers can design filtering cables with specific response characteristics by easily adjusting the seven stopbands, following the methodology described in this paper.

### Supplementary Information

Supplementary Information 1.

Supplementary Video 1.

## Data Availability

All data generated or analyzed during this study are included in this published article and its supplementary files. The datasets used and/or analyzed during the current study are available from the corresponding author on reasonable request.
