# Supplementary material for: Design of filtering cable with defected conductor layer
Source: Sci Rep. 2024 Mar 4;14:5227. doi: 10.1038/s41598-024-55736-9 (PMC10909839; doi:10.1038/s41598-024-55736-9)
Supplement: Supplementary file 1 — Supplementary Information 1. [file 41598_2024_55736_MOESM1_ESM.docx]

**Video. Simulated current distribution of the filter cable based on sawtooth dumbbell-shaped DCL.** (**a**) Surface current distribution on the DCL at 10 MHz within the passband. (**b**) Surface current distribution on the inner conductor at 10 MHz within the passband. (**c**) Surface current distribution on the DCL at 148 MHz within the stopband. (**d**) Surface current distribution on the inner conductor at 148 MHz within the stopband.
